# Genetic module and miRNome trait analyses reflect the distinct biological features of endothelial progenitor cells from different anatomic locations

**DOI:** 10.1186/1471-2164-13-447

**Published:** 2012-09-03

**Authors:** Cheng-Chung Cheng, Hung-Hao Lo, Tse-Shun Huang, Yi-Chieh Cheng, Shi-Ting Chang, Shing-Jyh Chang, Hsei-Wei Wang

**Affiliations:** 1 Division of Cardiology, Department of Internal Medicine, Tri-Service General Hospital, National Defense Medical Center, Taipei, Taiwan; 2 Institute of Microbiology and Immunology, National Yang-Ming University, Taipei, Taiwan; 3 Institute of Biomedical Informatics, National Yang-Ming University, No. 155, Sec 2, Li-Nong Street, Taipei, Taiwan; 4 Department of Obstetrics and Gynecology, Hsinchu Mackay Memorial Hospital, Hsinchu, Taiwan; 5 Department of Education and Medical Research, Taipei City Hospital, Taipei, Taiwan; 6 National Yang-Ming University VGH Genome Research Center, National Yang-Ming University, Taipei, Taiwan

## Abstract

**Background:**

Endothelial progenitor cells (EPCs) play a fundamental role in post-natal vascular repair, yet EPCs from different anatomic locations possess unique biological properties. The underlying mechanisms are unclear.

**Results:**

EPCs from CB expressed abundant genes involved in cell cycle, hypoxia signalling and blood vessel development, correlating with the phenotypes that CB-EPCs proliferated more rapidly, migrated faster, and formed tubule structure more efficiently. smRNA-seq further deciphered miRNome patterns in EPCs isolated from CB or PB: 54 miRNAs were enriched in CB-EPCs, while another 50 in PB-EPCs. Specifically, CB-EPCs expressed more angiogenic miRNAs such as miR-31, while PB-EPCs possessed more tumor suppressive miRNAs including miR-10a. Knocking down miR-31 levels in CB-EPCs suppressed cell migration and microtubule formation, while overexpressing miR-31 in PB-EPCs helped to recapitulate some of CB-EPC functions.

**Conclusions:**

Our results show the foundation for a more detailed understanding of EPCs from different anatomic sources. Stimulating the expression of angiogenic microRNAs or genes in EPCs of low activity (such as those from patients with cardiovascular diseases) might allow the development of novel therapeutic strategies.

## Background

The progressive impairment of endothelial function and integrity starts a cascade of events, leading to microcirculation damage, atherosclerosis and common cardiovascular disease (CVD), such as coronary heart disease (CHD), myocardial infarction (MI), heart failure, stroke and peripheral arterial disease (PAD) [1]. Blood-derived endothelial progenitor cells (EPCs) represent the “promoters” of vascular repair providing the rationale for autologous stem cell therapy [2]. The coexistence of multiple classical CVD risk factors negatively influences the number and functional activity of EPCs [3,4]. The number of EPCs has been reported to negatively correlate with hypertension, diabetes mellitus and aging but not smoking [5]. Levels of EPCs are inversely correlated to progression of coronary heart disease [6]. EPCs are currently being tested in different clinical settings including repair of damaged microcirculation, regeneration of ischemic tissues, and bioengineering of vascular grafts (http://www.clinicaltrials.gov/).

Clinically, circulating EPCs can be obtained from adult peripheral blood and umbilical cord blood. The number of EPCs in adult blood is known to be significantly lower than in cord blood [7]. EPCs derived from different anatomic locations, just like other somatic stem cells of different sources [8,9], possess unique biological activities: *in vitro* phenotypic studies demonstrated that CB-EPCs have competitive advantage compared with PB-EPCs due to their higher proliferative advantage, as well as better survival rate upon stress-induced apoptosis [10,11]. *In vivo*, tissue-engineered blood vessels generated by peripheral blood- and umbilical cord blood-derived EPCs: blood vessels formed by adult peripheral blood EPCs are unstable and regress within weeks, while umbilical cord blood EPCs form normal-functioning blood vessels that last for more than 4 months [11,12]. Thus, umbilical cord blood EPCs hold great therapeutic potential for cell therapy and vascular engineering.

The above findings suggest that CB-EPCs have enhanced vasculogenic ability compared with adult PB-EPCs. However, the underlying mechanisms are unclear. EPCs from human umbilical cord and adult peripheral blood activate different mechanisms upon high-dose x-ray radiation treatment: CB-EPCs undergo p53 stabilization, Bax-dependent apoptosis and p21-dependent G_1_ and G_2_/M cell cycle checkpoints, while PB-EPCs undergo only radiation-induced senescence [13], indicating unique gene expression patterns in EPCs of different sources. Another level of regulation may lie on microRNAs (miRNAs), which are endogenously expressed small non-coding RNAs of 18-24 nucleotides in length that regulate gene expression on the posttranscriptional level [14]. microRNAs have emerged as master regulators of stem cell lineage differentiation and angiogenesis [14]. microRNAs also play a crucial role in endothelial inflammation, senescence and susceptibility to atherosclerosis: endothelial inflammation is critically regulated by miRNAs such as miR-126 and miR-10a, and endothelial aging is additionally controlled by miR-217 and miR-34a [15]. miR-221 and miR-222, which are encoded from the same miRNA cluster, modulate the angiogenic properties of human umbilical vein endothelial cells (HUVECs) by targeting c-Kit and endothelial nitric oxide synthase (eNOS) [16]. In contrast, miRNA-31 enhances endothelial cell migration and invasion by targeting FAT4, a novel breast cancer tumor suppressor [17,18]. miR-126, -132, -296, -378, and the miR-17 ~ 92 cluster (encoding miR-17, -18a, -19a/b, -20a and miR-92a) also contribute to pathological angiogenesis [19-21].

In this study we explored protein-coding mRNAs and miRNAs involved in EPC activities. We found that CB-EPCs migrate faster and form tubule structures *in vitro* more efficiently than PB-EPCs do. mRNA and miRNA levels in EPCs of different origins reflect their unique performance.

## Methods

### Isolation and cultivation of EPCs

All patients gave informed consent, and the study was approved by the research ethics committee of the Hsinchu Mackay Memorial Hospital, Taiwan (ref number: 11MMHIS040). Protocols of this study were consistent with ethical guidelines provided in the 1975 Helsinki Declaration (http://www.wma.net/e/policy/b3.htm). EPC isolation and characterization were done as described previously with minor modifications [22,23]. In brief, blood mononuclear cells (MNCs) isolated by Histopaque-1077 (1.077 g/ml, Sigma, St. Louis, Missouri, USA) density-gradient centrifugation. MNCs (1 × 10^7^) were plated in 2 ml endothelial growth medium-2 (Lonza Ltd., Basel, Switzerland), with supplementation (hydrocortisone, IGF-1, human EGF, human VEGF, human FGF-B, ascorbic acid, GA-1000, heparin and 2% fetal bovine serum) on fibronectin-coated six-well plates at 37 °C in a 5% CO_2_ incubator. After 3 days of culturing, nonadherent cells were removed. Thereafter, the medium were replaced every 2 days, and EPCs colonies emerge 2–4 weeks after the start of MNC culture.

### EPC tube formation, transwell cell migration and cell proliferation assays

A miR-31 cDNA construct was used in overexpression experiments [18]. Tube formation assay was performed on EPCs to assess their capacity for vasculogenesis, which is believed to be important in new vessel formation. In brief, the *in vitro* tube formation assay was performed by thawing Matrigel at 4 °C overnight, and then placed it in a 96-well plate at 37 °C for 1 h to allow the matrix solution to solidify. EPCs were harvested with trypsin/EDTA, and 1 × 10^4^ EPCs were placed on Matrigel with EGM-2 medium or serum-free DMEM and incubated at 37 °C for 6 h. Tubule formation was inspected under an inverted light microscope (100x). Four representative fields were taken. For 3D angiogenesis assay, collagen type I acidic solution were mixed with 1/2 volume of basic conditioned medium with 0.2 ug/ml SDF-1α (R&D system, Minneapolis, MN USA) and solidify 30 minutes in 96-well plate at 37 °C in a 5% CO_2_ incubator. 10^5^ cells per well were seeded and assayed.

Cell migration ability was evaluated using Costar Transwell® Polycarbonate Permeable Supports (Corning, NY, USA) as previously described [18]. The degree of cell proliferation was examined by the MTT assay system (Invitrogen, USA) according to the manufacturer’s instructions.

### mRNA microarray and bioinformatics analysis

Total RNA sample preparation, cRNA probe preparation, array hybridization and data analysis were done as described previously [24]. Affymetrix^TM^ HG-U133 Plus 2.0 whole genome chips were used. RMA log expression units were calculated from Affymetrix GeneChip array data using the ‘affy’ package of the Bioconductor (http://www.bioconductor.org) suite of software for the R statistical programming language (http://www.r-project.org). The default RMA settings were used to background correct, normalize and summarize all expression values. Significant differences between the sample groups was identified using the ‘limma’ (Linear Models for Microarray Analysis) package of the Bioconductor suite, and an empirical Bayesian moderated t-statistic hypothesis test between the two specified phenotypic groups was performed [25]. To control for multiple testing errors, we then applied a false discovery rate algorithm to these *p* values in order to calculate a set of *q* values, thresholds of the expected proportion of false positives, or false rejections of the null hypothesis [26]. Heat maps were created by the dChip software (http://www.dchip.org/). Array data are deposited in the Gene Expression Omnibus (GEO) database with an accession number of GSE39763. Part of the PB EPC array data were from a public GEO dataset GSE23203 (GSM663476-81) and 1 CB-EPC data from GSE12891 (GSM323169).

Gene annotation was performed by our ArrayFusion web tool (http://microarray.ym.edu.tw/tools/arrayfusion/) [27]. Gene Ontology database search were performed by the DAVID 6.7 Bioinformatics Resources (http://david.abcc.ncifcrf.gov/). The Ingenuity Pathway Analysis (IPA) web tool developed by Ingenuity Co. (http://www.ingenuity.com) was used to construct functional regulatory networks of gene profiles. IPA uses the Ingenuity Pathways Knowledge Base to identify known interactions between focus genes and other genes that are not in the gene list. IPA then determines a statistical score for each network according to the fit of the network to the set of focus genes. The score is the negative log of p and denotes the likelihood of the focus genes in the network being found together by chance.

### Small RNA sequencing (smRNA-Seq) and data analysis

Total RNA was collected and small RNA fractions were sequenced by Illumina Solexa Genome Analyzer IIx (GAIIx; Illumina, San Diego, CA USA) according to manufacturer’s instruction. For data analysis, quality Fastq sequences, which were without poly-A, ambiguous nucleotides or a 5’ adapter, yet flanking 6-18 nt of 3’ adapter sequence, had the adapter sequences trimmed and the identical sequences were then collapse to unique sequences. The resulting unique sequences that did not align to mRNA database (UCSC genome browsers) but were aligned to known microRNA sequences (miRBase R18; http://www.mirbase.org/) were subjected into further quantification analysis. Sequencing reads were calculated to obtain a RPKM (reads per kilobase of exon model per million mapped reads) [28] value as C/LMN x 10^9^, where C = read numbers aligned to given miRNA chromosomal region, L = length of miRNA, M = multiple mapping numbers across all miRNA regions and N = total read numbers that map to human genome sequence. microRNA target prediction was done by the miRTar webtool (http://mirtar.mbc.nctu.edu.tw/human/) [29].

### RNA extraction and real-time quantitative polymerase chain reaction (qPCR)

RNA extraction and reverse transcription were performed as previously described [18]. The expression of mature human miRNAs was determined by a stem-loop real-time PCR system using the appropriate primer pairs. The universal PCR reverse primer for the miRNAs was 5’-GTGCAGGGTCCGAGGT-3’. miR-31-specific primers were used [18] and the primer sequences are in Additional file 1: Figure S1. Primer sequences of all other genes and miRNAs are also in Additional file 1*:* Figure S1. The miRNA expression data were normalized against the average values of U6 snRNA, U48 snRNA and 5S rRNA, while the miRNA expression data were normalized against the average values of GAPDH and beta-actin.

## Results

### Isolation and characterization of human EPCs from cord blood and adult peripheral blood

**Figure 1:**
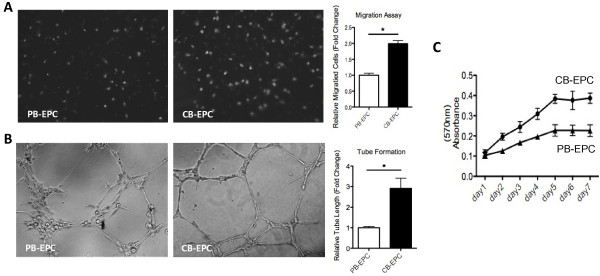
**Different angiogenic abilities between EPCs isolated from different anatomic locations.** ( **A**) EPCs from cord blood (CB-EPCs) migrate faster than those from adult peripheral blood (PB-EPCs). EPCs from different sources were subjected to Transwell cell migration assays, and migrated cells were stained (representative pictures are shown) and counted (left panel, n = 3). *:P < 0.05 ( **B**) CB-EPCs form better microvasculature structures *in vitro*. EPCs were subjected onto MatriGel for tube formation assays (representative pictures are shown). Tube lengths of formed microvascular structure were counted (left panel, n = 3). *:P < 0.05 ( **C**) Cell proliferation assays show CB-EPCs grow faster *in vitro*. Cultured EPCs were subjected into MTT assays for monitoring cell proliferation rate.

EPCs were obtained from cord blood or peripheral blood of healthy subjects as described [23]. Blood MNCs that were initially seeded on fibronectin-coated wells were round, and outgrowth EPCs with a cobblestone-like morphology similar to mature endothelial cells grew to confluence at days 14-21 (not shown). Cultured EPCs were subjected into Traswell cell migration assays (Figure 1A), tube formation assays (Figure 1B), or MTT assays (Figure 1C). Clearly EPCs from CB migrated faster, proliferated faster and formed microvasculature structure more efficient *in vitro* (Figure 1A-C).

### Gene expression signatures and functional modules of different EPCs

**Figure 2:**
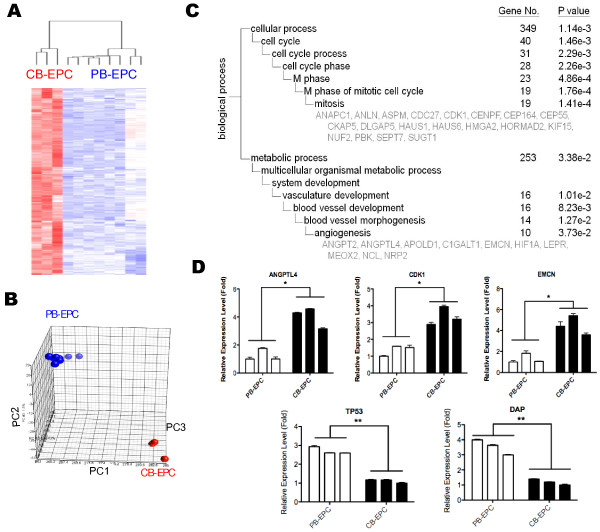
**Distinct gene expression patterns of different EPCs.** ( **A**) A heat map showing genes more abundant in CB-EPCs. Columns represent EPC samples from different donors, while rows represent probe sets. Genes in red: increased expression; in blue: decreased. ( **B**) A principle component analysis (PCA) plot using genes differentially expressed between different EPCs. Each spot represents a single array sample. ( **C**) The unique biological functions of CB-EPCs. CB-EPC-enriched genes were subjected to a Gene Ontology (GO) database search. These categories were selected from the “Biological Process” organizing principle in the GO database (http://www.geneontology.org/). The number of genes, gene symbols, and p values for each category that are significantly enriched are listed (p < 0.05). (**D**) Validation of mRNA array data by qPCR. Mean gene expression levels of EPC genes were compared to the average CT values of GAPDH and beta-actin controls. Results are expressed as mean ± standard deviation. *:P < 0.05 ( **E**) Canonical pathways enriched in CB-EPCs according to the analysis of the Ingenuity Pathway Analysis (IPA) web tool. ( **F**) Schematic representation of the “FLT3 Signaling in Hematopoietic Progenitor Cells” pathway. CB-EPC genes assigned to this pathway are indicated and in red.

To provide the underlying mechanisms for observed phenotypes, we explored the transcriptome patterns of different EPCs. Protein-coding mRNAs were deciphered first by Affymetrix whole-genome microarrays. A total of 753 probe sets (positive false discovery rate (pFDR) q < 0.005) were found unique to CB-EPC, while another 431 to PB-EPC (Figure 2A & Additional file 2*:* Figure S2 online). A PCA plot using these 1184 probe represents their differentiating power (Figure 2B).

The above gene list gave us a primary insight into the unique composition of differential EPCs but reflected little on EPC functions. To understand more how gene expression profiles might correlate with EPC biology and to provide quantitative evidence, signature probe sets were subjected to Gene Ontology (GO) database search for finding statistically over-represented functional groups within these genes. Given that the whole human transcriptome was represented by the microarray analysis, this analysis was not biased toward the coverage of the microarray. The GO categories of the biological processes being statistically overrepresented (*p* < 0.05) among CB-EPC genes are presented in Figure 2C. The most significant biological process for CB-EPCs is cell cycle (349 genes, *p* = 1.14 × 10^-3^), especially the mitotic cell cycle (19 genes, *p* = 1.41 × 10^-4^; Figure 2C). Vasculature development genes (16 genes, *p* = 1.01 × 10^-2^), especially those involved in angiogenesis (10 genes, *p* = 3.73 × 10^-2^), are also significantly higher in CB-EPCs (Figure 2C). The abundant expression of CB-EPC or PB-EPC genes were verified by RT-qPCR (Figure 2D). A famous tumor suppressor TP53 was more abundant in PB-EPC, while angiogenic genes ANGPTL4 and CDK1 in CB-EPCs (Figure 2D). Other related predominant processes include those pertaining to DNA damage checkpoint (8 genes, *p* = 5380 × 10^-4^, not shown), protein transport (38 genes, *p* = 0.0034), and post-translational protein modification (53 genes, *p* = 0.0045, especially those involve in phosphorylation (37 genes, *p* = 0.0124)).

We also subjected CB-EPC genes into KEGG and Ingenuity Pathway Analysis (IPA) database search for disclosing enriched pathways and functional modules. More information was revealed from Ingenuity database search. The “G2/M DNA damage checkpoint regulation” canonical pathway ranks the No. 1 most significant pathway found among CB-EPC genes (Figure 2E). Genes involved in the “FLT3 signaling in hematopoietic progenitor cells” pathway is also overexpresssed in CB-EPCs (Figures 2E-F). Also enriched in CB-EPCs are HIF1α (hypoxia-inducible transcription factor 1 alpha) signaling, cardiac hypertrophy signaling, renin-angiotensin signaling and NFAT in cardiac hypertrophy pathways (Figure 2E), reflecting the pro-angiogenic nature of CB-EPCs. By KEGG definition, genes involved in cell cycle are again found significant (Additional file 3*:* Figure S3). Database search and functional module assays explain in part why CB-EPCs amplification quicker (Figure 1C).

### Unique miRNA expression profiles of different EPCs revealed by small RNA sequencing (smRNA-Seq)

**Figure 3:**
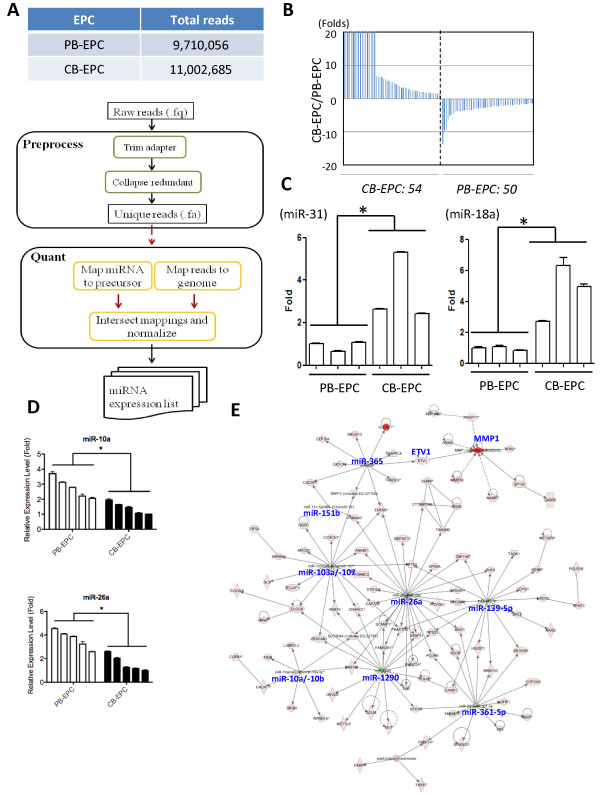
**Differentially expressed miRNAs between CB- and PB-EPCs discovered by smRNA-Seq.** ( **A**) A table summarizes reads number ( *upper*) and a flowchart describes the data analysis pipeline for quantification of known miRNAs from smRNA-Seq data ( *lower*). ( **B**) Differential expressed miRNAs between cord blood EPCs and adult peripheral blood EPCs. ( **C-D**) qPCR validation of smRNA-Seq data. Mean miRNA expression levels were compared to the average CT values of U6 snRNA + U48 snRNA + 5S rRNA controls. miRNAs more abundant in CB-EPC ( **C**) or PB-EPC ( **D**) were verified. *:P < 0.05 ( **E**) A major functional genetic network composed of multiple PB-EPC microRNAs (in green) and CB-EPC genes (in red). This network is displayed graphically as nodes (gene products) and edges (biological relationships between nodes) mapped by the Ingenuity Pathway Analysis (IPA) tool. The intensity of the node color indicates the degree of differential expression. Hub miRNAs in this genetic network are shown.

Another level of gene expression regulation is through microRNAs. To provide a more comprehensive view of transcriptome profiles of EPCs from different sources, we determined miRNA profiles of different EPCs by sequencing the small RNA fractions of both EPCs. Illumina Solexa platform generated 9.7 million high-quality sequence reads for PB-EPCs, and another 11 million reads for CB-EPCs (Figure 3A, *upper*). We constructed an in-house pipeline (illustrated in Figure 3A) for analyzing sequencing data. The initial operations included identifying sequence matches to the mRNA database in order to eliminate degraded mRNA exon reads. Then non-exonic reads that match previously annotated miRNAs deposited in the miRBase database (release 18) were subjected to normalization and quantitative profiling. The expression of known miRNAs were converted into RPKM, and then filtered using a threshold RPKM > 100. A total of 104 miRNAs were differentially expressed between 2 EPCs, with 54 being more abundant in CB-EPCs while another 50 in PB-EPCs (≥1.5 folds; Figure 3B & Tables 1-2). The differential expression of miR-31, miR-18a, miR-10a and miR-26a were verified by RT-qPCR (Figures 3C-D).

**Table 1:** 54 miRNAs over-expressed in CB-EPC

**Name**	**chromosome location**	**PB RPKM**	**CB RPKM**	**Fold (CB/PB)**	**PB rank**	**CB rank**
hsa-mir-136	chr14:101351053-101351075	0	495.64316	9.90E+307	770	95
hsa-mir-376c	chr14:101506069-101506089	1.2448007	1174.4226	943.4623551	483	68
hsa-mir-494	chr14:101496018-101496039	3.3418648	3083.2217	922.6051575	382	33
hsa-mir-376a*	chr14:101507125-101507146	3.1190746	2104.9521	674.8643011	389	47
hsa-mir-376a	chr14:101506455-101506475	0.7002004	359.68243	513.6849822	542	106
	chr14:101507162-101507182					
hsa-mir-377	chr14:101528431-101528452	0.89116406	305.87003	343.2252755	514	116
hsa-mir-410	chr14:101532298-101532318	1.4004008	399.5301	285.2969664	468	101
hsa-mir-381	chr14:101512305-101512326	6.6837296	1888.779	282.5935687	316	54
hsa-mir-411	chr14:101489677-101489697	5.134803	1427.5367	278.0119705	341	63
hsa-mir-889	chr14:101514286-101514306	1.4004008	309.84427	221.2539939	468	113
hsa-mir-379	chr14:101488408-101488428	27.541208	4422.998	160.5956427	221	27
hsa-mir-369-3p	chr14:101531978-101531998	5.6016035	868.6908	155.0789519	334	81
hsa-mir-134	chr14:101521031-101521052	10.9167595	910.0818	83.36556283	281	77
hsa-mir-29b	chr7:130562226-130562248	84.74453	6061.3887	71.52542707	162	20
	chr1:207975795-207975817					
hsa-mir-222*	chrX:45606479-45606500	25.843756	1562.2592	60.45016057	223	61
hsa-mir-31	chr9:21512157-21512177	347.53278	15717.314	45.22541442	93	7
hsa-mir-127-3p	chr14:101349372-101349393	64.60941	2614.9524	40.47324376	174	39
hsa-mir-654-3p	chr14:101506606-101506627	16.932117	517.527	30.5648136	255	91
hsa-mir-146a	chr5:159912379-159912400	1061.7109	7229.664	6.809446903	65	15
hsa-mir-216a	chr2:56216155-56216176	462.73697	3054.6147	6.601190089	84	34
hsa-mir-18b	chrX:133304114-133304136	95.57735	621.04553	6.497831652	152	90
hsa-mir-24-2*	chr19:13947140-13947161	126.99086	810.7064	6.383974406	144	84
hsa-mir-503	chrX:133680401-133680423	83.323845	498.72934	5.985433581	163	94
hsa-mir-18a	chr13:92003010-92003032	116.24848	684.41486	5.887516637	147	87
hsa-mir-4792	chr3:24562903-24562920	56.91072	308.37952	5.418654341	181	114
hsa-mir-19a	chr13:92003193-92003215	168.31697	886.21814	5.265174034	130	80
hsa-mir-19b	chrX:133303713-133303735,	351.0184	1759.4749	5.01248624	92	58
	chr13:92003499-92003521					
hsa-mir-185	chr22:20020676-20020697	130.77832	639.70306	4.891506941	142	89
hsa-mir-424	chrX:133680710-133680731	326.61172	1557.3126	4.768085481	97	62
hsa-mir-24	chr9:97848346-97848367	39987.26	172634.36	4.317234039	5	2
	chr19:13947103-13947124					
hsa-mir-196a	chr17:46709894-46709915	93.497955	398.9361	4.266789578	156	102
	chr12:54385546-54385567					
hsa-mir-130a	chr11:57408725-57408746	319.70517	1143.3563	3.576283424	98	70
hsa-mir-345	chr14:100774213-100774234	137.23924	455.79385	3.321162737	138	98
hsa-mir-32	chr9:111808552-111808573	94.68617	312.1079	3.296235343	153	112
hsa-mir-339-3p	chr7:1062591-1062613	116.35501	376.72174	3.237692472	146	105
hsa-mir-186	chr1:71533364-71533385	1265.8989	3930.9248	3.105243871	57	30
hsa-mir-20b	chrX:133303880-133303902	393.07117	1119.9288	2.849175634	90	72
hsa-mir-542-3p	chrX:133675394-133675415	302.77307	758.6524	2.505679914	100	85
hsa-mir-877	chr6:30552109-30552128	134.05331	333.61777	2.488694759	140	110
hsa-mir-106b	chr7:99691666-99691686	1116.7422	2731.2852	2.445761609	62	36
hsa-mir-452	chrX:151128150-151128171	466.9701	1105.1763	2.366696069	82	73
hsa-mir-22	chr17:1617208-1617229	1037.9835	2326.5046	2.24136954	66	42
hsa-mir-374a*	chrX:73507130-73507151	1247.1844	2619.4692	2.100306258	60	38
hsa-mir-29c	chr1:207975210-207975231	1326.5715	2675.448	2.01681402	53	37
hsa-mir-29a	chr7:130561507-130561528	1443.6847	2802.1428	1.940965919	50	35
hsa-mir-30e	chr1:41220043-41220064	5007.227	9227.503	1.842836963	26	10
hsa-mir-99a	chr21:17911421-17911442	2773.0786	5025.056	1.812085673	41	24
hsa-mir-20a	chr13:92003326-92003348	1287.8253	2302.079	1.787570876	54	43
hsa-mir-15a	chr13:50623303-50623324	483.79068	822.4292	1.699969086	81	83
hsa-mir-100	chr11:122022983-122023004	4361.801	7380.455	1.692065961	28	14
hsa-mir-106a	chrX:133304274-133304296	1250.3193	2036.8033	1.629026521	59	50
hsa-mir-17	chr13:92002872-92002894	1266.1599	2058.4065	1.625708175	56	48
hsa-mir-27a	chr19:13947261-13947281	5321.171	8077.8022	1.51804973	25	12
hsa-mir-140-5p	chr16:69967006-69967027	334.1866	501.82477	1.501630436	96	93

**Table 2:** 50 miRNAs over-expressed in PB-EPC

**Name**	**chromosome location**	**PB RPKM**	**CB RPKM**	**Fold (CB/PB)**	**PB rank**	**CB rank**
hsa-let-7b*	chr22:46509625-46509646	358.35938	26.206163	-13.67462226	91	280
hsa-mir-15b*	chr3:160122433-160122454	695.9995	72.2731	-9.630132096	73	215
hsa-mir-1290	chr1:19223572-19223590	337.0359	35.74031	-9.43013365	95	258
hsa-mir-15b	chr3:160122395-160122416	2379.7402	379.11118	-6.27715648	42	104
hsa-mir-574-3p	chr4:38869713-38869734	435.55652	86.03938	-5.062292639	85	201
hsa-mir-25	chr7:99691194-99691215	1534.8059	319.06284	-4.810356167	49	111
hsa-mir-148a	chr7:25989542-25989563	1073.8528	231.33844	-4.641912516	63	134
hsa-mir-30e*	chr1:41220085-41220106	3634.1675	920.4779	-3.948131183	34	76
hsa-mir-30a*	chr6:72113257-72113278	4051.6772	1061.4753	-3.817024475	29	74
hsa-mir-365	chr16:14403197-14403218,	653.44617	171.86374	-3.802117713	75	149
	chr17:29902497-29902518					
hsa-mir-28-3p	chr3:188406622-188406643	1804.1597	485.26227	-3.717906401	44	96
hsa-mir-23b	chr9:97847547-97847567	8076.4937	2225.3198	-3.629363159	22	45
hsa-mir-146b-5p	chr10:104196277-104196298	1253.9794	349.96533	-3.583153223	58	108
hsa-mir-92b	chr1:155165028-155165049	318.21985	90.19796	-3.528016044	99	195
hsa-mir-197	chr1:110141562-110141583	528.68317	149.92363	-3.526349849	79	156
hsa-mir-140-3p	chr16:69967045-69967065	12527.033	3577.7405	-3.501381109	12	32
hsa-mir-598	chr8:10892731-10892752	489.91757	143.68575	-3.40964619	80	161
hsa-let-7b	chr22:46509571-46509592	8703.284	2554.5288	-3.407001714	20	40
hsa-mir-378	chr5:149112430-149112450	32406.646	10804.972	-2.999234612	7	9
hsa-mir-455-3p	chr9:116971767-116971787	548.25714	187.93459	-2.917276378	78	142
hsa-mir-193b	chr16:14397874-14397895	415.28253	143.25558	-2.898892525	87	162
hsa-mir-92a	chrX:133303574-133303595	3847.525	1331.7456	-2.889084071	33	64
	chr13:92003615-92003636					
hsa-mir-93	chr7:99691438-99691460	3588.8904	1322.95	-2.712793681	35	65
hsa-let-7c	chr21:17912158-17912179	15608.78	5758.0083	-2.710794981	11	22
hsa-mir-30b	chr8:135812813-135812834	1066.1298	400.58517	-2.661431026	64	100
hsa-mir-10a	chr17:46657266-46657288	17177.37	6494.583	-2.644876507	8	17
hsa-mir-10b	chr2:177015057-177015079	16707.91	6317.509	-2.644699042	10	19
hsa-let-7d	chr9:96941123-96941144	9057.177	3642.2817	-2.48667669	16	31
hsa-mir-151-3p	chr8:141742686-141742706	17066.672	7218.801	-2.3641976	9	16
hsa-mir-30c	chr1:41222972-41222994	4914.325	2188.866	-2.245146574	27	46
	chr6:72086706-72086728					
hsa-let-7e	chr19:52196046-52196067	8924.559	3985.3586	-2.239336505	17	29
hsa-mir-320a	chr8:22102488-22102509	757.8811	340.61673	-2.225026058	70	109
hsa-let-7a	chr11:122017276-122017297	65673.34	30296.24	-2.16770596	3	5
	chr9:96938244-96938265					
	chr22:46508632-46508653					
hsa-mir-99b	chr19:52195871-52195892	8875.32	4099.9907	-2.164717105	19	28
hsa-let-7f	chr9:96938635-96938656	37668.82	17587.143	-2.141838501	6	6
	chrX:53584207-53584228					
hsa-mir-23a	chr19:13947409-13947429	10271.966	4879.2725	-2.105224908	14	26
hsa-mir-125b	chr21:17962573-17962594	3983.9465	1976.1088	-2.016056252	30	51
	chr11:121970517-121970538					
hsa-mir-125a-5p	chr19:52196521-52196544	1764.7084	897.535	-1.966172238	46	79
hsa-mir-361-5p	chrX:85158686-85158707	399.24158	205.20401	-1.945583714	89	138
hsa-mir-16	chr3:160122542-160122563	3338.2983	1761.0107	-1.895671787	38	57
	chr13:50623163-50623184					
hsa-mir-1307	chr10:105154058-105154079	553.6359	294.46994	-1.880110072	77	118
hsa-mir-320b	chr1:224444751-224444772	661.5061	356.97238	-1.853101632	74	107
	chr1:117214409-117214430					
hsa-mir-217	chr2:56210155-56210177	10865.332	5983.509	-1.815879612	13	21
hsa-mir-769-5p	chr19:46522219-46522240	421.96628	236.17818	-1.786643796	86	133
hsa-mir-191	chr3:49058105-49058127	3378.5552	1927.2273	-1.753065246	36	52
hsa-mir-139-5p	chr11:72326147-72326168	301.21347	178.96194	-1.683114689	101	145
hsa-mir-26a	chr12:58218441-58218462	3344.9814	2055.193	-1.627575318	37	49
	chr3:38010904-38010925					
hsa-mir-103a	chr5:167987909-167987931	8117.7095	5296.3853	-1.532688624	21	23
	chr20:3898188-3898210					
hsa-mir-107	chr10:91352513-91352535	3862.0547	2533.423	-1.524441319	31	41
hsa-mir-151b	chr14:100575775-100575792	610.3607	405.608	-1.504804392	76	99

Applying the genetic network analysis function in the IPA web tool, we searched for miRNA-mRNA pairs and networks in CB-EPCs. PB-EPC miRNAs, such as miR-10a/b, miR-26a, miR-103a, miR-107, miR-139-5p, miR-151b, miR-361-5p, miR-365 and miR-1290, were found to be master regulators of a subset of CB-EPC protein-coding mRNAs (Figure 3E). The collective reduction of these miRNAs in CB-EPCs may explain in part why genes, such as ETV1 (Figure 3E), in this genetic network are more abundant in CB-EPCs.

Most of the differentially expressed miRNAs have not been linked to angiogenesis. MiR-410 is involved in regulating secretion [30]. MiR-15a, -20b and -24 are reduced in the plasma of type 2 diabetes patients, which intend to have poor angiogenesis [31]. *In vitro*, miR-503 expression in ECs is upregulated in culture conditions mimicking diabetes mellitus (high D-glucose) and ischemia-associated starvation (low growth factors). Forced miR-503 expression inhibits EC proliferation, migration, and network formation on Matrigel [32]. MiR-24 is considerably upregulated after cardiac ischemia and is enriched in cardiac endothelial cells. MiR-24 induces endothelial cell apoptosis, abolishes endothelial capillary network formation on Matrigel, and inhibits cell sprouting from endothelial spheroids by targeting of the stemness transcription factor GATA2 and the p21-activated kinase PAK4 [33,34]. MiR-100 has an antiangiogenic function by modulating proliferation, tube formation, and sprouting activity of endothelial cells and migration of vascular smooth muscle cells and functions as an endogenous repressor of the serine/threonine protein kinase mammalian target of rapamycin (mTOR) [35]. MiR-29b can suppress tumor angiogenesis, invasion and metastasis by regulating MMP-2 expression in hepatocellular carcinoma (HCC) [36]. MicroRNAs from the miR-17 ~ 92 cluster are known to contribute in pathological angiogenesis [19-21], and 5 out of 6 members (including miR-17, -18a, -19a/b and -20a) were overexpressed in CB-EPCs (Table 1).

### miR-31 as a novel EPC angiogenic miRNA

**Figure 4:**
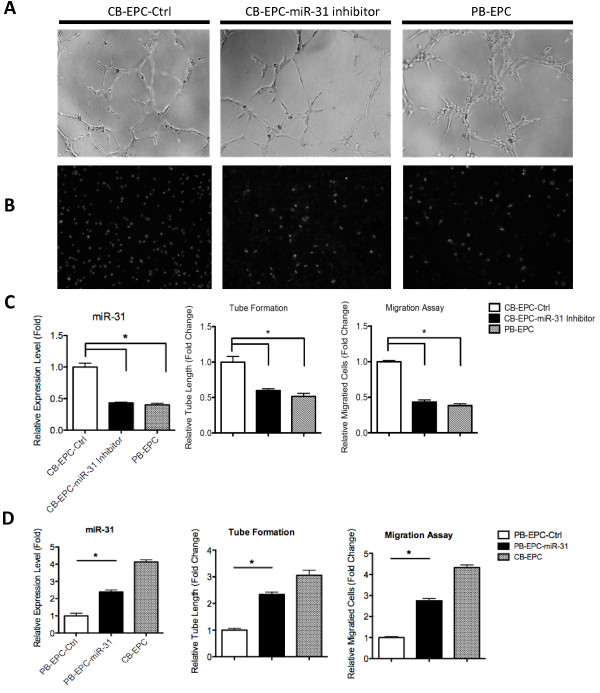
**miR-31 is involved in EPC activities.** ( **A-C**) Knocking down endogenous miR-31 in CB-EPCs. Anti-miR-31 antagomiR or the siGFP control (Ctrl) was introduced into CB-EPCs by electroporation, and 2 days later EPCs were subjected to tube formation ( *A*) and Transwell cell migration assays ( *B*). Cellular miR-31 levels were detected by RT-qPCR ( *C*, left panel; n = 3), and migration assay and tube formation assay results were also quantified ( *C*, middle and right panels; n = 3, using cells from 3 batches of donors). ( **D**) Overexpressing miR-31 in PB-EPCs stimulates EPC angiogenic abilities. Cellular miR-31 levels were detected by RT-qPCR (left panel; n = 3). Cellular migration assays and tube formation assays were done, and results were quantified (middle and right panels; n = 3, using cells from 3 batches of donors).

To identify more pro-angiogenic miRNAs involved in EPC activity, we examined which miRNA(s) may contribute in EPC angiogenesis. MiR-31 is a known pro-angiogenic and pro-lymphangiogenic miRNA which induce motility in both matured blood vessel and lymphatic endothelial cells [18,37]. Knocking down endogenous miR-31 levels reduced tube formation and cellular migration abilities in CB-EPCs (Figures 4A-C), suggesting a pro-angiogenic role of miR-31 in both progenitor and mature type endothelial lineage cells. On the other hand, overexpressing miR-31 in PB-EPCs helped to recapitulate some of the functions of CB-EPCs (Figure 4D). Over-expressing miR-31 in PB-EPC or knocking down endogenous CB-EPC miR-31 level did not affect cell proliferation rate at a significant level in the first 24 hours of transfection (Additional file 4*:* Figure S4). The tube formation and cell migration effects we observed should due to mainly the pro-angiogenic activity of miR-31.

## Discussion

Endothelial cells from the internal barrier of the vasculature, and play fundamental roles in vascular development and disease. The regulation of angiogenesis depends not only on the number of circulating EPC but also on their functions [38]. Aberrant EPC activity and the resulting abnormal angiogenesis cause a variety of diseases, such as ischemia, cancer and metastasis. On the other hand, these cells are also potential cell source for cellular therapies aiming to enhance the neovascularization of tissue engineered constructs or ischemic tissues [39]. Atherosclerotic heart disease remains one of the major causes of morbidity and mortality worldwide. Currently, vascular revascularization techniques, including balloon angioplasty and stenting, have been well developed. However, post-angioplasty restenosis substantially limit long-term benefits of heart revascularization procedures. Therapeutic progenitor cell transplantation bear potential for organ vascularization regeneration in various pathological states [40]. The application of EPC in stenting technology during vascular revascularization is the Genous EPC stent (OrbusNeich, Wanchai, Hong Kong), which is a stainless-steel stent coated with immobilized human anti-CD34 monoclonal antibodies that allow the stent surface to "capture" EPCs in the blood to accelerate endothelialization of the stent strut. In recent clinical trial, it shows promise result in reducing the risk of stent thrombosis by facilitating rapid endothelialization on stent strut [41].

An increasing number of studies shows that miRNAs, or angiomiRs, play a crucial role in regulating various aspects of cancer biology, including angiogenesis. Manipulating miRNAs in the settings of pathological vascularization therefore represents a new therapeutic approach [14]. On the other hand, there are still challenges to harnessing EPCs for cell therapy. One of these is their rarity (0.01-0.02 per 10^6^ mononuclear cells), which makes EPC isolation challenging. *In vitro* cultivation and amplification of EPCs is therefore required before these cells may be appropriately investigated for use in clinical therapies. However, it is crucial to maintain EPC activity during such *in vitro* manipulation. Understanding the basic EPC biology will help to develop new biomarkers for monitoring EPC activities. In this report, we identified EPCs, especially those from cord blood, exploit several cellular genetic groups and miRNA pathways to regulate their angiogenesis activities. Transcriptome information will eventually help to develop new clinical applications as mentioned above.

When PB-EPC genes were subjected into GO database search, we found these genes are enriched in both Wnt receptor signaling (8 genes including CREBBP, DVL3, NFAT5, PPARD, RAC1, TBL1X, TCF7L2, TP53; p= 0.019) and positive regulation of I-kappaB kinase/NF-kappaB cascade (6 genes including LITAF, MAP3K3, MAP3K7IP2, MUL1, TRIM13, PSMB7; p = 0.048). Genes involved in the induction of apoptosis are also more abundant in PB-EPCs (15 genes, p = 0.006). KEGG and IPA database search also revealed that genes involved in both Wnt signaling (p = 0.020) and MAPK (13 genes, including ACVR1B, ARRB1, DDIT3, DUSP3, DUSP16, ELK4, GADD45B, MAP3K3, MAP3K7IP1, MAP3K7IP2, MAPKAPK2, RAC1 and TP53; p = 012) pathways are more abundant in PB-EPCs (Additional file 5*:* Figure S5, Additional file 6*:* Table S1). IPA analysis also revealed the Wnt/β-catenin signaling pathway may be more active in PB-EPCs (Additional file 7*:* Table S2). The Wnt signaling system regulates vascular patterning in the developing embryo [42]. It has recently been documented that Wnt1 is a proangiogenic molecule of human endothelial progenitor function, and increases blood flow to ischemic limbs in a HGF-dependent manner [43]. Our work further supports a crucial role of Wnt pathway in adult EPCs, and Wnt proteins may be therapeutically deployed to increase blood flow and angiogenesis in adult ischemic tissues.

In this study we applied RNA sequencing (RNA-seq) technology, instead of microRNA chips, for deciphering EPC miRNomes. This is due to the fact that microarray application in miRNome research has several limitations, including hybridization and cross-hybridization artifacts, dye-based detection issues and design constraints that preclude or seriously limit the detection of newly discovered miRNAs or previously unmapped, novel miRNAs [44]. These issues have made it difficult for standard array designs to provide full sequence comprehensiveness (coverage of all possible genes, including “unknown” ones, in large genomes) or transcriptome comprehensiveness (reliable detection of all RNAs of all prevalence classes, including the least abundant ones that are physiologically relevant). Studies using this method have already altered our view of the extent and complexity of eukaryotic transcriptomes [44]. RNA-seq has also delivered a sharp rise in the rate of novel microRNA discovery in the current miRBase R18 release (2011 Nov; http://microrna.sanger.ac.uk/sequences/), which is the primary online repository for all microRNA sequences and annotation.

One of the CB-EPC-dominant microRNAs is miR-31, a pro-angiogenic miRNA that enhances endothelial cell migration [18,37]. MiR-31 has recently been documented as a signature BEC miRNA that negatively regulates lymphatic endothelial cell identity and lymphatic vascular development by targeting Prox1, a transcription factor that functions as a master regulator of lymphatic lineage-specific differentiation [45]. In the present study, we further showed that miR-31 is a dominant miRNA in CB-EPCs, and its overexpression is crucial for EPCs to possess superior angiogenic ability (Figure 4). Unmasking the roles of small RNA-mediated gene regulation in EPC activity will be crucial and will provide new insights into regenerative and reparative medicine. We envision that our report will serve as a resource for future miRNA studies that aim to improve understanding of the various regulatory ultimately modulating EPC and EC activities.

For miRNAs more abundant in PB-EPCs, miR-217 modulates endothelial cell senescence via silent information regulator 1 (sirT1) [46]. The levels of miR-330 and let-7e are higher in the myocardial microvascular endothelial cells (MMVEC) in type 2 diabetic Goto-Kakizaki (GK) rats, which have impaired angiogenesis [47]. MiR-93, member of the miR-106b ∼ 25 cluster (a paralog of the miR-17 ∼ 92 cluster), in tumor cells possesses oncogenic and angiogenic activities [48]. Both microRNA-125a-5p and miR-125b are overexpressed in PB-EPCs (Table 2), and their role in inhibiting endothelin-1 expression in vascular endothelial cells has been reported [49]. miR-10a regulates a proinflammatory phenotype in athero-susceptible endothelium *in vivo*[50]. The collective effects of these miRNAs in EPC biology are still awaited to be elucidated.

## Conclusions

In summary, our results reveal a series of differentially expressed miRNAs and protein-coding genes that have not previously been associated with EPC biology. This study therefore provides a road map for future mechanistic studies of EPC migration and microvasculature formation, which should eventually help to improve our understanding of angiogenesis, and will also benefit the development of new therapeutic approaches that target the inhibition of pathogenic angiogenesis in tumors, the stimulation of angiogenesis in patients with cardiovascular diseases, stroke or diabetes.

## Competing interests

The authors declare no competing financial interests.

## Authors’ contributions

C-CC and H-HL carried out the study design, performed the statistical analysis and drafted the manuscript. H-H L and Y-CCcarried out the cell biology assays. T-SH and S-TC participated in sequence alignment. S-JC provided clinical samples. H-WW conceived of the study, and participated in its design and coordination and helped to draft the manuscript. All authors read and approved the final manuscript.

## Supplementary Material

Additional file 1 Figure S1 Primers used in RT-qPCR validation. Click here for file

Additional file 2 Figure S2 Gene expression signatures and functional modules of different EPCs. Click here for file

Additional file 3 Figure S3 Distribution of CB-EPC cell cycle genes according to the KEGG database. CB-EPC genes are labeled with red stars. The P value is also shown. Click here for file

Additional file 4 Figure S4 Over-expressing miR-31 in PB-EPC (A) or knocking down endogenous miR-31 in CB-EPC (B) did not affect cell proliferation rate at a significant level in the first 24 hours of transfection. Click here for file

Additional file 5 Figure S5: Distribution of PB-EPC genes in the Wnt signaling pathway according to the KEGG database. PB-EPC genes are labeled with red stars. Click here for file

Additional file 6 Table S1 Distribution of PB-EPC genes in the MAPK signaling pathway according to the KEGG database. PB-EPC genes are labeled with red stars. Click here for file

Additional file 7 Table S2 Distribution of PB-EPC genes in the Wnt signaling pathway according to the IPA web tool. Involved PB-EPC genes are in green and indicated. Click here for file
