# Supplementary material for: Genetic module and miRNome trait analyses reflect the distinct biological features of endothelial progenitor cells from different anatomic locations
Source: BMC Genomics. 2012 Sep 3;13:447. doi: 10.1186/1471-2164-13-447 (PMC3443421; doi:10.1186/1471-2164-13-447)
Supplement: Additional file 1 Figure S1 — Primers used in RT-qPCR validation. [file 1471-2164-13-447-S1.pdf]

Suppl. Table 1. Primers used in RT-qPCR validation

	Forward	Reverse
**U6 snRNA**	5' CGCTTCGGCAGCACATATAC 3'	5' TTCACGAATTTGCGTGTCAT 3'
**U48 snRNA**	5' AGTGATGATGACCCCAGGTAACTC 3'	5' CTGCGGTGATGGCATCAG 3'
**5S rRNA**	5' GCCCGATCTCGTCTGATCT 3'	5' CAAGTACTAACCAGGCCCGA 3'
**β-Actin**	5' GGCATCCTCACCCTGAAGTA 3'	5' AGGTGTGGTGCCAGATTTTC 3'
**ANGPTL4**	5' GGCAGAGTGGACTATTTGAAATCC 3'	5' TTACTGTCCAGCCTCCATCTGA 3'
**CDK1**	5' TGGAGAAGGTACCTATGGAGTTGTG 3'	5' CCCTTCCTCTTCACTTTCTAGTCTGA 3'
**EMCN**	5' CCTAAAGGAACAATCACCAATGAAT 3'	5' GGCTTTCAATCCTTCATCTTTACTTG 3'
**TP53**	5’ CATTCTGGGACAGCCAAGTC 3’	5’ AATCAACCCACAGCTGCAC 5’
**DAP**	5’ CCTCCCGAAGGGAAACTAGA 3’	5’ CTTTGGTGTCTCCTGTATGTGG 3

	Forward	RT stem-loop primer
**miR-31**	5' GGAGAGGCAAGATGCTGGCA 3'	5' GTCGTATCCAGTGCAGGGTCCGAGGTATTCGCACTGGATACGACAGCTAT 3'
**miR-26a**	5’ CGCGCTTCAAGTAATCCAGG 3’	5’ GTCGTATCCAGTGCAGGGTCCGAGGTATTCGCACTGGATACGACAGCCTA 3’
**miR-10a**	5’ GGCGTACCCTGTAGATCCGAA 3’	5' GTCGTATCCAGTGCAGGGTCCGAGGTATTCGCACTGGATACGACCACAAA 3’
