# Supplementary figures and images for: Genetic module and miRNome trait analyses reflect the distinct biological features of endothelial progenitor cells from different anatomic locations
Source: BMC Genomics. 2012 Sep 3;13:447. doi: 10.1186/1471-2164-13-447 (PMC3443421; doi:10.1186/1471-2164-13-447)

## Slide 1
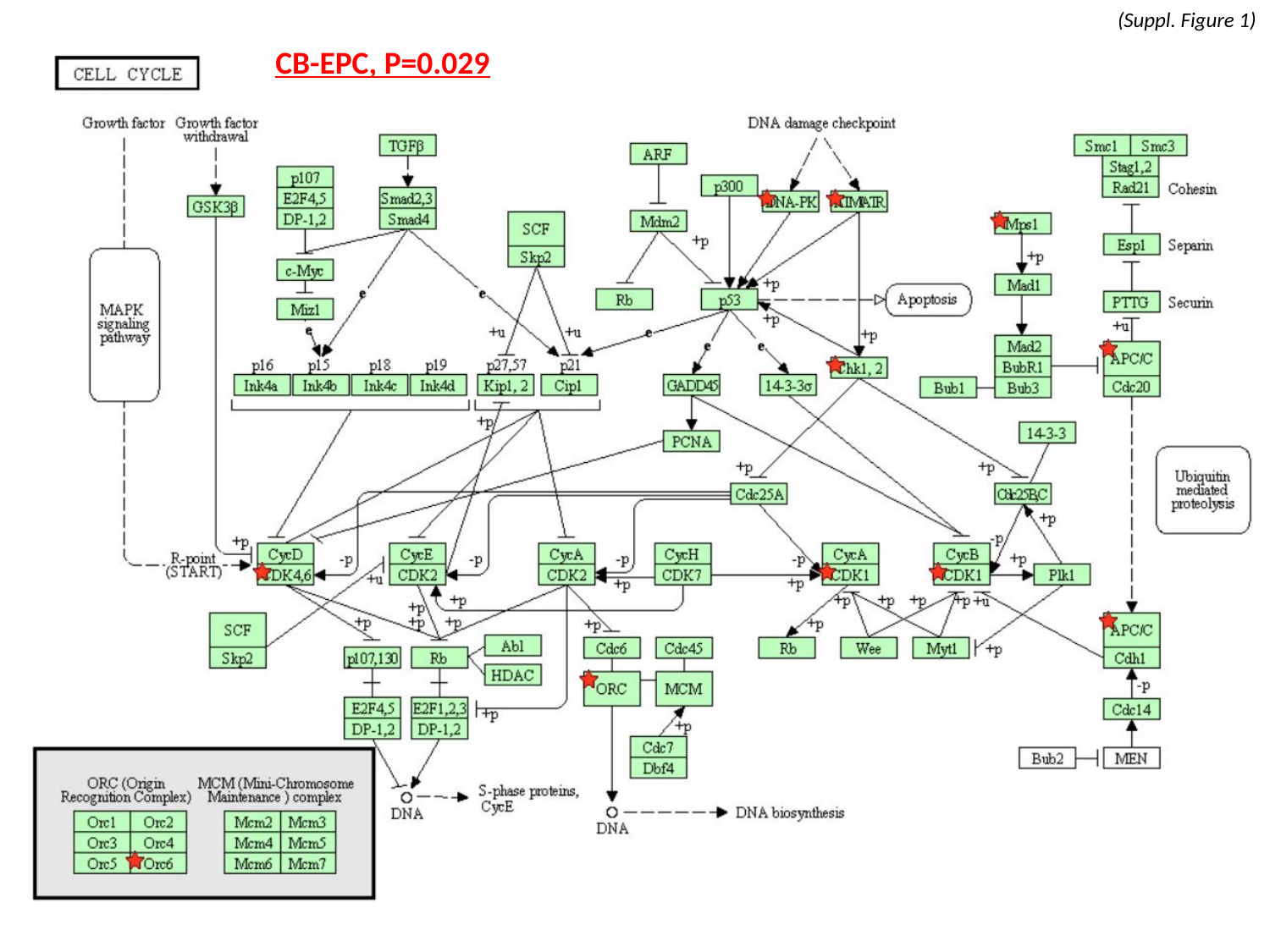

(Suppl. Figure 1)
CB-EPC, P=0.029

Supplement: Additional file 3 Figure S3 — Distribution of CB-EPC cell cycle genes according to the KEGG database. CB-EPC genes are labeled with red stars. The P value is also shown. [file 1471-2164-13-447-S3.ppt]

## Slide 1
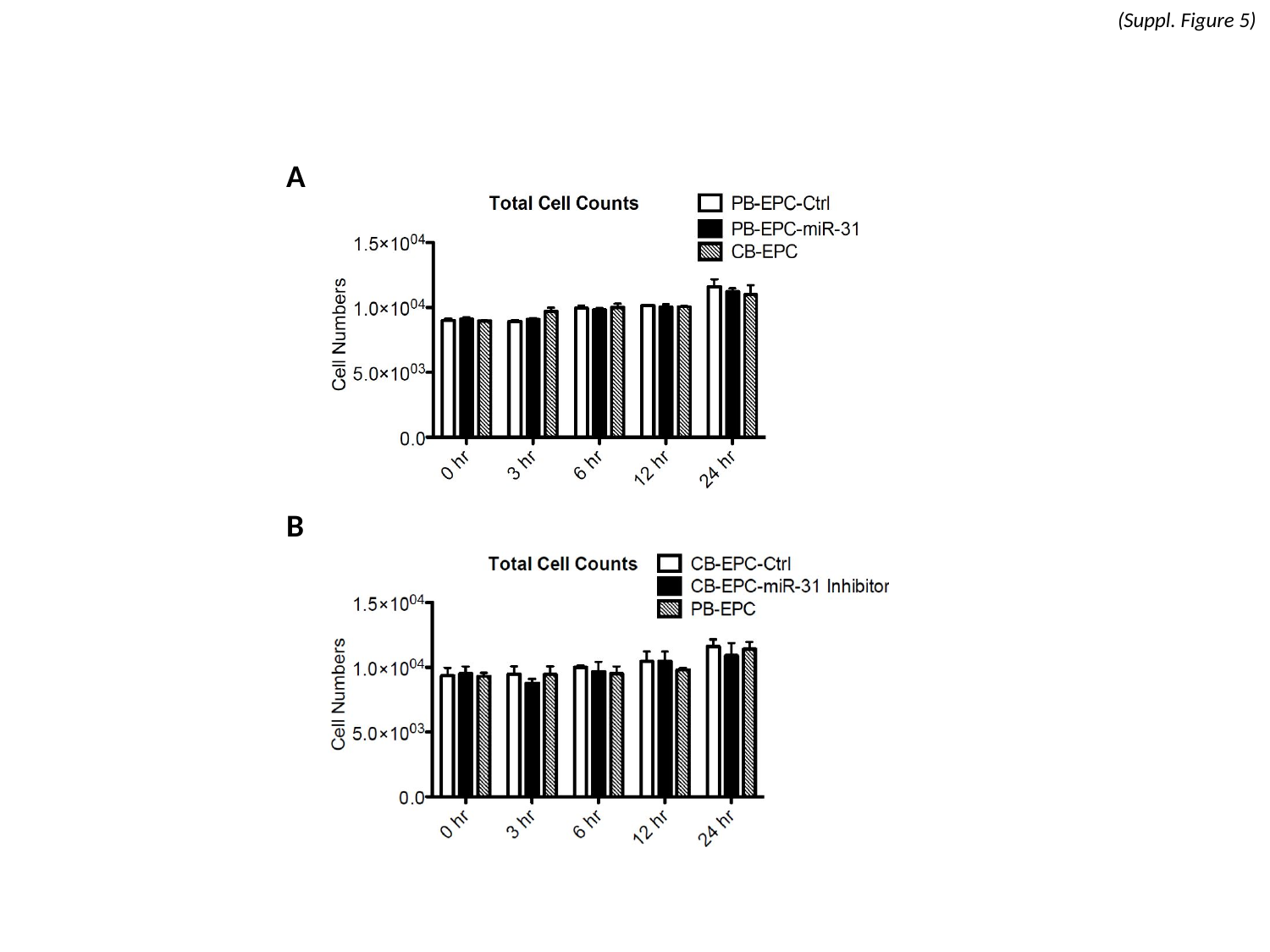

(Suppl. Figure 5)
A
B

Supplement: Additional file 4 Figure S4 — Over-expressing miR-31 in PB-EPC (A) or knocking down endogenous miR-31 in CB-EPC (B) did not affect cell proliferation rate at a significant level in the first 24 hours of transfection. [file 1471-2164-13-447-S4.ppt]

## Slide 1
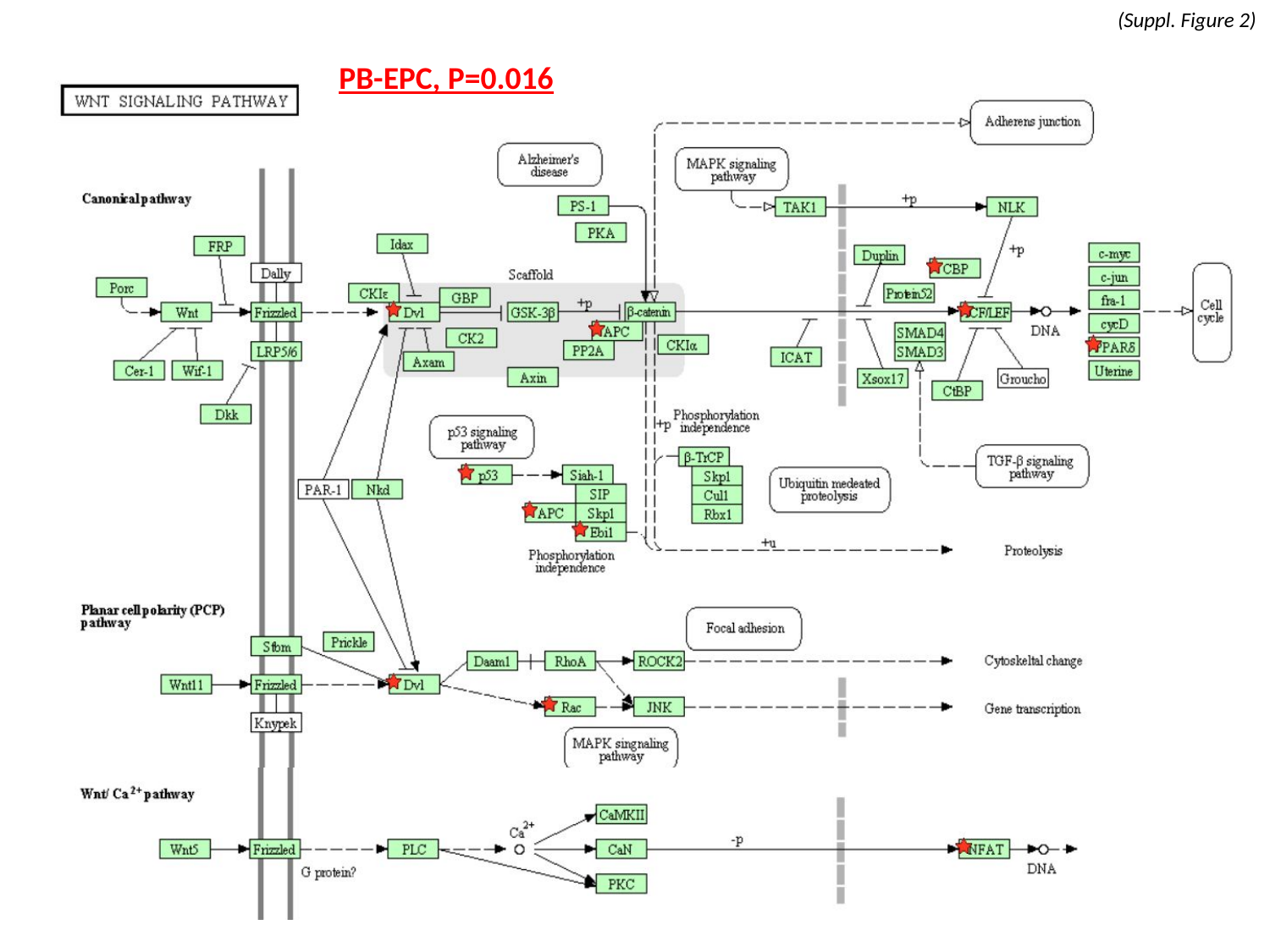

(Suppl. Figure 2)
PB-EPC, P=0.016

Supplement: Additional file 5 Figure S5: — Distribution of PB-EPC genes in the Wnt signaling pathway according to the KEGG database. PB-EPC genes are labeled with red stars. [file 1471-2164-13-447-S5.ppt]

## Slide 1
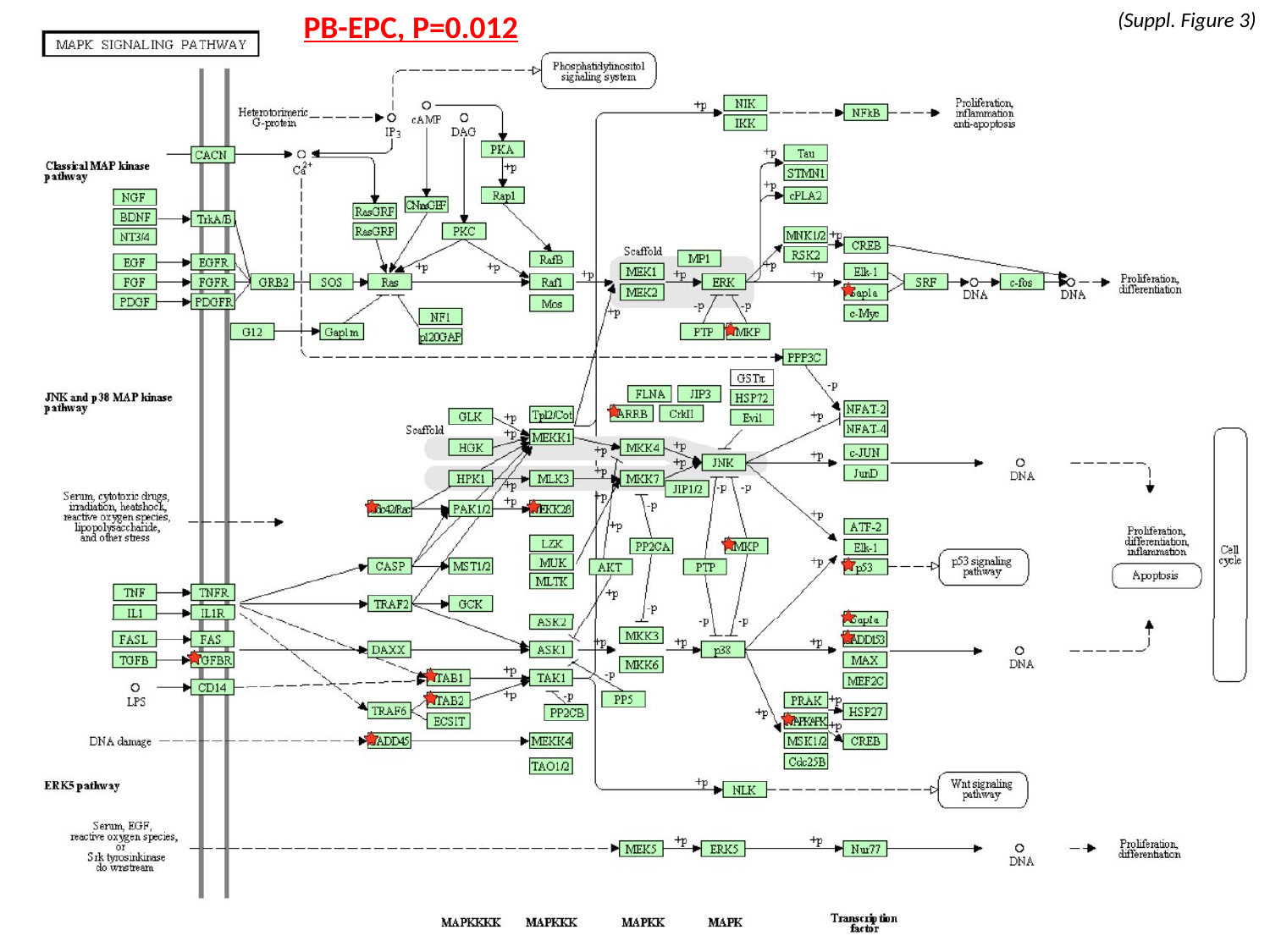

PB-EPC, P=0.012
(Suppl. Figure 3)

Supplement: Additional file 6 Table S1 — Distribution of PB-EPC genes in the MAPK signaling pathway according to the KEGG database. PB-EPC genes are labeled with red stars. [file 1471-2164-13-447-S6.ppt]

## Slide 1
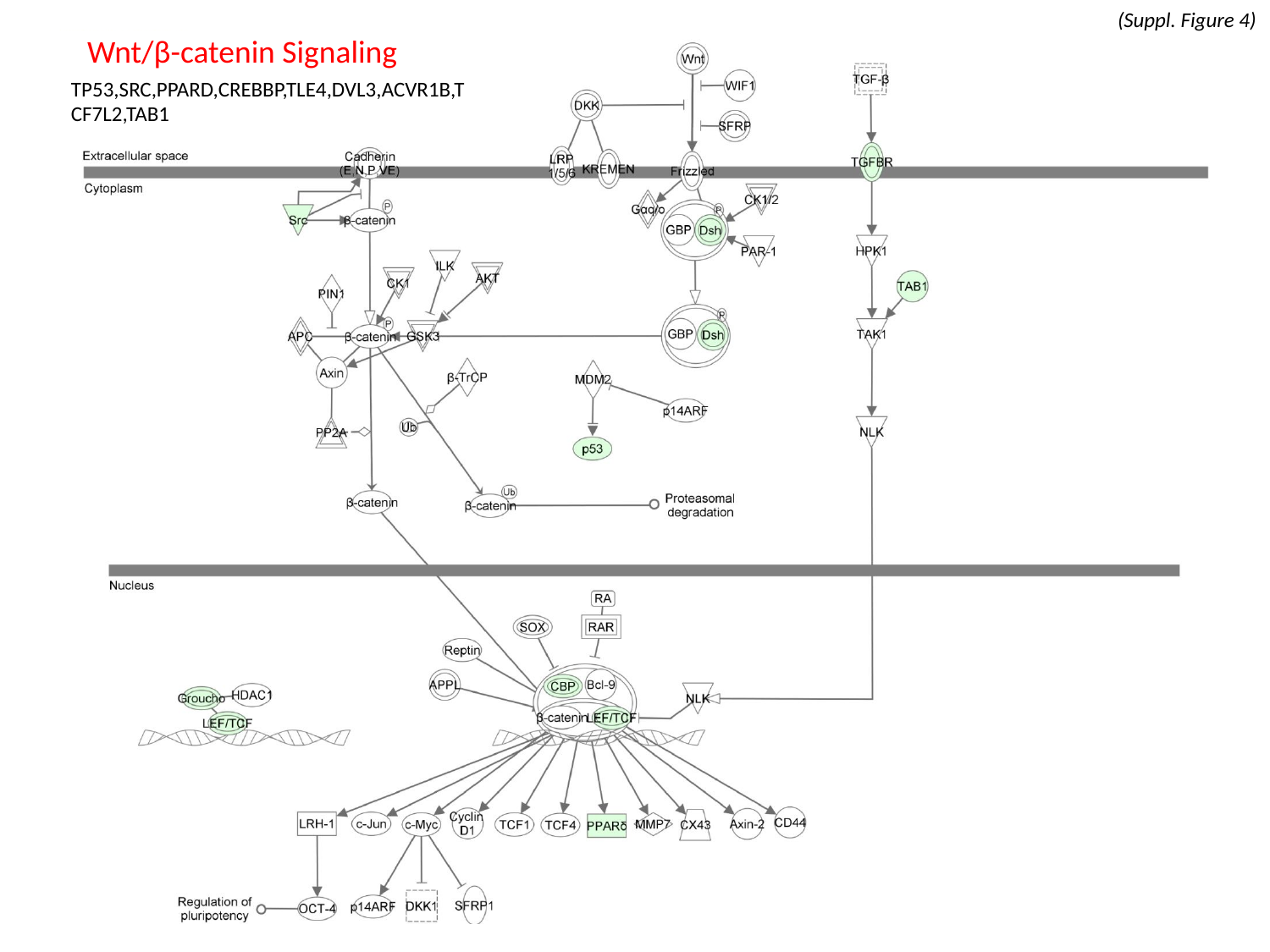

(Suppl. Figure 4)
Wnt/β-catenin Signaling
TP53,SRC,PPARD,CREBBP,TLE4,DVL3,ACVR1B,TCF7L2,TAB1

Supplement: Additional file 7 Table S2 — Distribution of PB-EPC genes in the Wnt signaling pathway according to the IPA web tool. Involved PB-EPC genes are in green and indicated. [file 1471-2164-13-447-S7.ppt]
